# Ghrelin Modulates the fMRI BOLD Response of Homeostatic and Hedonic Brain Centers Regulating Energy Balance in the Rat

**DOI:** 10.1371/journal.pone.0097651

**Published:** 2014-05-15

**Authors:** Miklós Sárvári, Pál Kocsis, Levente Deli, Dávid Gajári, Szabolcs Dávid, Zsófia Pozsgay, Nikolett Hegedűs, Károly Tihanyi, Zsolt Liposits

**Affiliations:** 1 Laboratory of Endocrine Neurobiology, Institute of Experimental Medicine, Hungarian Academy of Sciences, Budapest, Hungary; 2 Preclinical Imaging Center, Gedeon Richter Ltd., Budapest, Hungary; 3 Faculty of Information Technology, Pázmány Péter Catholic University, Budapest, Hungary; Institut d'Investigacions Biomèdiques August Pi i Sunyer, Spain

## Abstract

The orexigenic gut-brain peptide, ghrelin and its G-protein coupled receptor, the growth hormone secretagogue receptor 1a (GHS-R1A) are pivotal regulators of hypothalamic feeding centers and reward processing neuronal circuits of the brain. These systems operate in a cooperative manner and receive a wide array of neuronal hormone/transmitter messages and metabolic signals. Functional magnetic resonance imaging was employed in the current study to map BOLD responses to ghrelin in different brain regions with special reference on homeostatic and hedonic regulatory centers of energy balance. Experimental groups involved male, ovariectomized female and ovariectomized estradiol-replaced rats. Putative modulation of ghrelin signaling by endocannabinoids was also studied. Ghrelin-evoked effects were calculated as mean of the BOLD responses 30 minutes after administration. In the male rat, ghrelin evoked a slowly decreasing BOLD response in all studied regions of interest (ROI) within the limbic system. This effect was antagonized by pretreatment with GHS-R1A antagonist JMV2959. The comparison of ghrelin effects in the presence or absence of JMV2959 in individual ROIs revealed significant changes in the prefrontal cortex, nucleus accumbens of the telencephalon, and also within hypothalamic centers like the lateral hypothalamus, ventromedial nucleus, paraventricular nucleus and suprachiasmatic nucleus. In the female rat, the ghrelin effects were almost identical to those observed in males. Ovariectomy and chronic estradiol replacement had no effect on the BOLD response. Inhibition of the endocannabinoid signaling by rimonabant significantly attenuated the response of the nucleus accumbens and septum. In summary, ghrelin can modulate hypothalamic and mesolimbic structures controlling energy balance in both sexes. The endocannabinoid signaling system contributes to the manifestation of ghrelin's BOLD effect in a region specific manner. In females, the estradiol *milieu* does not influence the BOLD response to ghrelin.

## Introduction

The hunger signal ghrelin [1] is a potent orexigenic hormone [2] that acts via its G-protein coupled receptor, GHS-R1A [3] within the central nervous system. The expression of GHS-R1A mRNA shows a discrete albeit wide distribution pattern within the neuroaxis [4], [5]. The preganglionic neurons of parasympathetic centers in the brainstem, hypothalamic nuclei with longstanding history of contribution to regulation of feeding and energy expenditure, the hippocampal formation and the dopaminergic mesostriatal and mesolimbic systems arising from the rostral mesencephalon are primary targets of direct, receptor-mediated signaling of ghrelin. The binding of radiolabeled ghrelin to identical, GHS-R1A containing loci proves the expression and functional capacity of the receptor protein [6]. In addition to the stomach [7], which provides the major ghrelin supply for the brain, the production of ghrelin in a discrete set of hypothalamic neurons has also been shown [8]. Ghrelin-immunoreactive axons have been detected in rodent [8], [9] and *post-mortem* human [10] brains.

Although ghrelin signaling regulates several systems and modulates a wide array of physiological functions in the organism [11], [12], a special attention follows its role in the regulation of feeding and energy expenditure [13]–[16]. Pioneer works have elucidated that ghrelin targets the orexigenic neuropeptide Y/agouti-related protein-synthesizing neuron population in the medial aspect of the arcuate nucleus [1], [8], [17] that, in turn, relays the orexigenic signal to other hypothalamic feeding centers including the paraventricular nucleus and the lateral hypothalamus [18]. They can respond to the hunger signal and execute effector commands via the endocrine and autonomic systems. These ghrelin-regulated mechanisms ensure the homeostatic control over feeding and energy expenditure [19]. In more recent years, an intriguing concept has emerged about the explicit role of ghrelin in regulation of the reward system [20]–[24]. Pharmacological targeting of the laterodorsal tegmentum-ventral tegmental area-mesolimbic system axis by GHS-R1A agonists and/or antagonists has shown the involvement of ghrelin in shaping the hedonic component in the control of energy balance [25]. Ghrelin seems to increase the incentive value of rewarding foods [23], [26], [27], alcohol [28] and abusing drugs [29], [30]. It also triggers the cascades of reward seeking behavior [31]. Antagonizing ghrelin signaling centrally offers a challenging approach in the fight against obesity, substance and eating disorders [28], [32], [33].

A fascinating finding has been that ghrelin modifies the activity of targeted neurons [8], [34] and modulates the incoming information of their synaptic afferents. Molecular events downstream from GHS-R1A have not been fully explored yet. A prevailing role has been attributed to PI3K-Akt [35] and AMPK [36] signaling mechanisms at cellular level. Recent studies have revealed that the sirtuin 1/p53 pathway links GHS-R1A activation and AMPK phosphorylation in the hypothalamus [37]. Besides PI3K-Akt and AMPK, additional mechanisms are involved in ghrelin action including the κ-opioid pathway in the hypothalamus [38]. Furthermore, increasing number of evidence indicates that GHS-R1A activation results in changes of endocannabinoid levels in the target cell that, in turn, modify the synaptic input of neuronal afferents via retrograde signaling [39]. Accordingly, CB1 receptors and intact retrograde endocannabinoid signaling mechanisms have been reported essential in the mediation of the orexigenic action of ghrelin in the paraventricular nucleus of the rat [39], and also in the modulation of firing by ghrelin in gonadotropin-releasing hormone (GnRH) neurons of mice [40]. In addition to the cooperation of ghrelin and endocannabinoid signaling systems, the modulatory role of the gonadal hormone 17β-estradiol (E2) has also been raised. Males and females with low E2 levels show a higher response to ghrelin in terms of food intake and body weight gain compared to females with high E2 levels [41], although a recent study has demonstrated no difference in food intake and the signaling pathway downstream to GHS-R1A in response to ghrelin in the two sexes [42].

In the present study, we focused on mapping BOLD responses evoked by ghrelin in the male and female rat brains and on elucidation of the role of endocannabinoid and E2 signaling in the process with special attention to brain centers controlling energy balance.

## Materials and Methods

### Ethics Statement

All studies were carried out with permission from the Animal Welfare Committee of the Institute of Experimental Medicine, Hungarian Academy of Sciences (Permission Number: A5769-01) and in accordance with the legal requirements of the European Community (Decree 86/609/EEC). In all studies, animal experimentation was conducted in accord with accepted standards of animal care.

### Reagents

Rat ghrelin was obtained from Tocris (Bristol, UK). Rimonabant was synthesized by the Synthetic Laboratory of Gedeon Richter Plc (Budapest, Hungary). GHS-R1A antagonist JMV2959 was a kind gift from Aeterna Zentaris GmbH (Frankfurt, Germany).

### Animals

Male (n = 20) and female (n = 24) Wistar rats, weighing 240–260 and 170–190 g, respectively, were purchased from Harlan. Rats were housed in light (12∶12 light-dark cycle, lights on at 6am) and temperature (21±1°C) controlled environment, with free access to standard food (sniff R/M+H Spezieldiäten GmbH D-59494 Soest) and tap water. The females were surgically ovariectomized (OVX) and half of them were replaced with E2 (OVX+E2) for 10 days as described elsewhere [43]. The average weight of OVX controls (n = 12) and E2 replaced animals (n = 11) was 219.2 g and 207.6 g, respectively. The weight of OVX controls was significantly larger (p = 0.031).

### MRI Experiments

Functional MRI experiments were performed on a 9.4T ASZ Varian MRI system with a free bore of 210 mm, containing a 120 mm inner size gradient coil (180 µs rise time). For excitation, an actively RF-decoupled 2 channel volume coil system with inner size 72 mm was used and a fix tuned receive-only phase array rat brain coil located directly above the dorsal surface of the animal's head to maximize the signal to noise ratio.

Scout pictures were obtained in planes of coronal and sagittal in order to set the anatomical and functional images. Anatomical scans were acquired using gradient echo multi slice (GEMS) sequence with a field of view, FOV 35×35 mm, slice thickness 1 mm, gap 0.2 mm. Nine slices were received in interleaved order; the scanner's default coronal orientation was slightly changed to get a standard anatomically coronal plane according to the Rat Brain Atlas of Paxinos & Watson [44]. Echo time, TE = 3.83 ms, repetition time, TR = 200 ms, flip angle 45°, averages 3, dummy scans 4, data matrix 192×192, total scan time 2 min.

An interleaved triple-shot gradient-echo echo planar imaging, EPI sequence with compressed segments was used for T2*-weighted MR images. TE = 10 ms, TR = 3000 ms, flip angle 90°, averages 1, dummy scans 4, data matrix 64×64, 1000 repetitions. FOV and slice parameters were the same as in the anatomical setup.

Rats were anaesthetized with isoflurane (5% starting concentration and then 1–1.5% during scanning) administered in compressed air. For intravenous drug administration, a cannula line was inserted and used during the scanning. The anesthetized rat was transferred into the magnet. Body temperature was monitored using a rectal probe and maintained at 37 ± 1°C via a thermostatically controlled air, flowed around the rat. The ventilation was also controlled.

Experiments lasted for 50 min. After 16 min 40 sec (1000 sec) baseline period 20 µg ghrelin was administered i.v. The automated drug administration was performed with an infusion pump controlled by optical signals. GHS-R1 antagonist JMV2959 (6 mg/kg) and rimonabant (3 mg/kg) or were applied i.p. to conscious animals as a pretreatment one hour prior to ghrelin administration. One measurement was performed with each animal. The results of each measurement were stored in the scanner's own file format which were converted to the widely used nifti-format (Neuroimaging Informatics Technology Initiative) using a Matlab script. More detailed technical information about the MRI has been published earlier [45].

### Data Analysis

Data analysis was performed as described previously [45]. In brief, displacement was checked and measurements with higher than one voxel movement were excluded. For creating t-maps in the phMRI experiments, paired t-test was performed on each voxel's two time intervals (pre-injection baseline and post-injection) to evaluate the difference between the baseline signal and post-injection signal. A t-value was ordered to every voxels and voxels over the t value limit were highlighted. Region of interest (ROI) analysis was performed using a Matlab script. ROIs were determined according to the Rat Brain Atlas of Paxinos & Watson [44]. Statistical significance of drug effect was evaluated by multifactorial ANOVA followed by *post-hoc* Fisher test.

### List of ROIs and Their Abbreviations

We analyzed the following areas: amygdala (Amyg), arcuate nucleus (ARC), cerebellum (cb), dorsomedial nucleus (DMH), hippocampus (Hipp), lateral hypothalamus (LH), lateral parabrachial nucleus (LPB), laterodorsal tegmental nucleus (LDTg), nucleus accumbens (Acb), nucleus tractus solitarii (Sol), paraventricular nucleus (PVN), parietal associative cortex (PAC), prefrontal cortex (PFC), septum (Sept), striatum (Str), substantia nigra (SN), suprachiasmatic nucleus (SCN), temporal cortex (TC), ventral tegmental area (VTA), ventromedial nucleus (VMH).

## Results

### BOLD Response to Ghrelin in Homeostatic and Hedonic Brain Centers Regulating Energy Balance in the Male Rat

**Figure 1:**
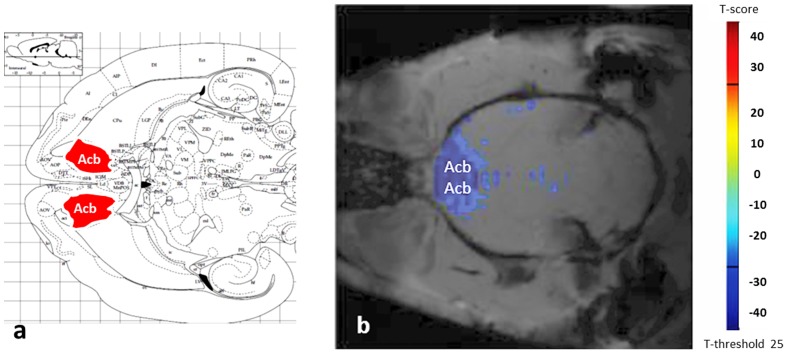
Characteristic fMRI BOLD response to ghrelin in the nucleus accumbens. **a**. Position of the nucleus accumbens (highlighted in red) is shown in a horizontal scheme of the rat brain [44]. **b**. Ghrelin evoked a negative BOLD response in the nucleus accumbens. Slice thickness is 1 mm.

In the first line of the experiment, the effect of ghrelin (20 µg, i.v.) was tested in either saline or GHS-R1A antagonist JMV2959 (6 mg/kg, i.p.) pretreated male rats. The applied dose of ghrelin corresponds to 25 nM/kg, which induces a marked stimulatory effect in plasma GH levels [46]. ROIs were divided into three groups, each containing well-defined nuclei and anatomical areas as follows: Group I. Reward processing neuronal assembly including the ventral tegmental area, nucleus accumbens, the prefrontal cortex, amygdala, septum and hippocampus. Representative fMRI image showing the nucleus accumbens was presented (Fig. 1). The temporal cortex and associative parietal cortex were included as controls. Group II. Homeostatic feeding centers of the hypothalamus were represented by the arcuate nucleus, ventromedial nucleus, dorsomedial nucleus, paraventricular nucleus, lateral hypothalamus and the circadian rhythm generator suprachiasmatic nucleus. Group III. Brainstem areas regulating hedonic and homeostatic mechanisms of energy balance included the laterodorsal tegmental nucleus, lateral parabrachial nucleus, nucleus tractus solitarii. In addition, we analyzed the main components of the nigrostriatal projection, the substantia nigra and the striatum, and also the 9–10th lobules of cerebellum.

**Figure 2:**
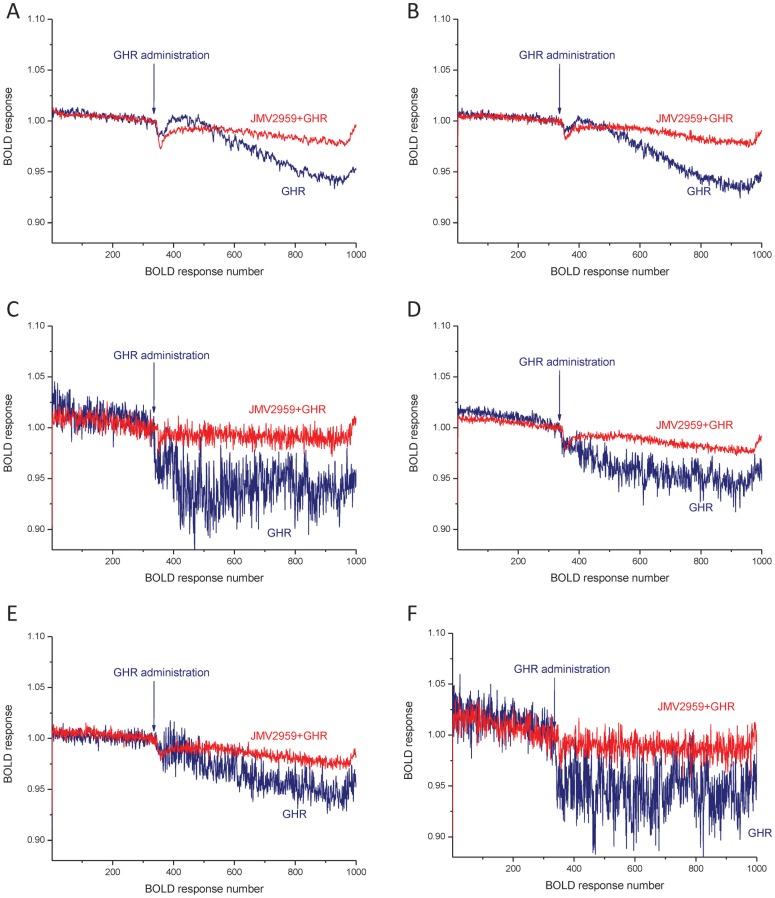
Mean of time response curves of ghrelin's effect on BOLD responses in the prefrontal cortex (A), nucleus accumbens (B), ventromedial nucleus (C), lateral hypothalamus (D), paraventricular nucleus (E) and suprachiasmatic nucleus (F). Blue and red colors indicate pretreatment with saline and GHS-R1A antagonist (JMV2959), respectively. Arrows mark ghrelin (GHR) administration, which started at BOLD response number 333 and lasted to 383 (999–1149 s). For quantifying drug effect, mean of the BOLD responses from 901 to 950 was calculated (2703–2850 s). N = 5–7.

**Figure 3:**
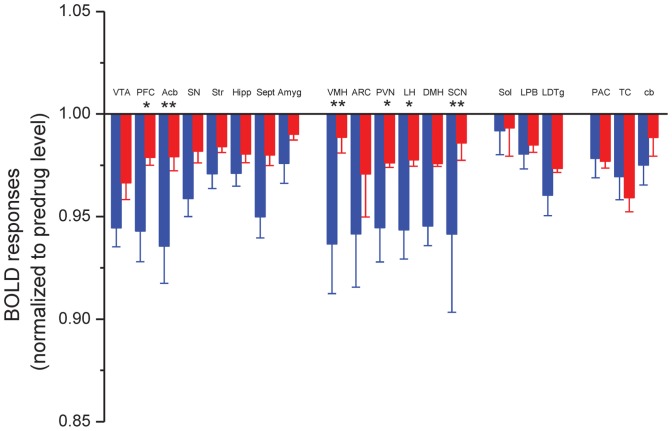
Effect of ghrelin on BOLD responses in reward processing centers, hypothalamic nuclei, brainstem and control areas of the rat brain. The effect of ghrelin after vehicle pretreatment is represented by blue columns and after JMV2959 pretreatment in red columns. Statistically significant differences were found in the prefrontal cortex (PFC), nucleus accumbens (Acb), lateral hypothalamus (LH), paraventricular nucleus (PVN), ventromedial nucleus (VMH) and suprachiasmatic nucleus (SCN). ANOVA and Fisher *post hoc* test *: p<0.05; **: p<0.01. N = 5–7.

We found a widespread regional response to ghrelin in hedonic and homeostatic centers. The evoked BOLD responses differed from each other in the saline and JMV2959 pretreated groups (Fig. 2). In general, ghrelin decreased the BOLD response in several loci of the neuroaxis, while JMV2959 pretreatment attenuated this effect (Fig. 3). In Group I, the prefrontal cortex (Fig. 2A) and the nucleus accumbens (Fig. 2B) showed the highest response to ghrelin, and also a powerful and significant counteracting effect of JMV2959. Moderate effects of ghrelin were observed in the ventral tegmental area, the hippocampus, septum and the amygdala (Fig. 3). In these loci, the BOLD response was not significant. In Group II, representing the main hypothalamic feeding centers and their modulator systems, ghrelin was also potent in decreasing the BOLD response. Among these structures, the ventromedial nucleus (Fig. 2C), the lateral hypothalamus (Fig. 2D), the paraventricular nucleus (Fig. 2E) and the suprachiasmatic nucleus (Fig. 2F) demonstrated significant BOLD responses. The primary feeding center of the hypothalamus, the arcuate nucleus also displayed a marked response to ghrelin which was attenuated with the antagonist pretreatment (Fig. 3). In Group III, the laterodorsal tegmental nucleus, the lateral parabrachial nucleus, the nucleus tractus solitarii showed weak, non-significant BOLD responses (Fig. 3). A similar activation pattern characterized the additionally examined regions like the striatum, the parietal, temporal cortices and the cerebellar cortex (Fig. 3).

### Role of Sex and Estradiol in the Ghrelin-Evoked BOLD Response

**Figure 4:**
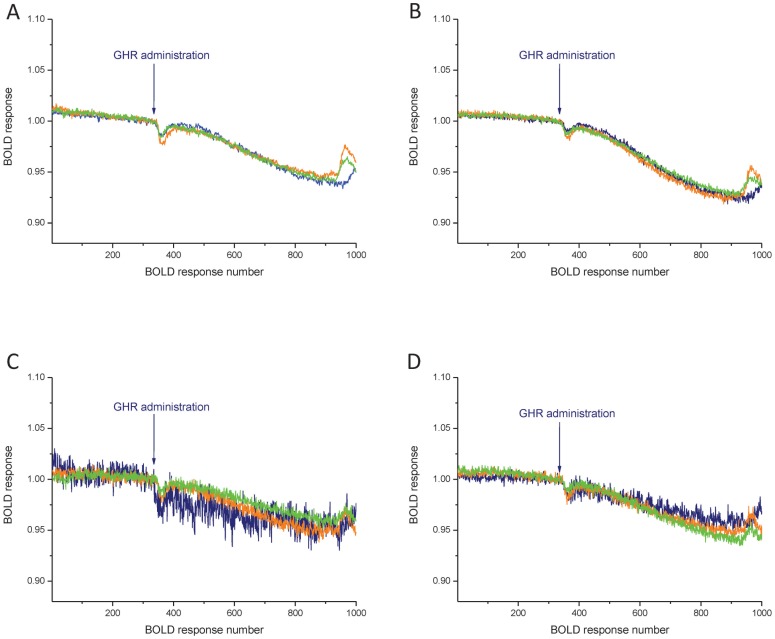
Mean of time response curves of ghrelin's effect on the BOLD responses in the prefrontal cortex (A), nucleus accumbens (B), ventromedial nucleus (C) and paraventricular nucleus (D), in males (blue), ovariectomized females (orange) and ovariectomized females treated with E2 (green). Arrows mark ghrelin administration, which started at BOLD response 333 and lasted to 383 (999–1149 s). For quantifying drug effect mean of the BOLD responses from 901 to 950 was calculated (2703–2850 s). N = 11–14.

In addition to the male, fMRI studies have also been carried out in chronically ovariectomized (OVX) and ovariectomized E2 replaced (OVX+E2) female rats in order to reveal the putative role of sex and the actual E2 *milieu* in the generation of the BOLD response evoked by ghrelin. The examined sets of ROIs were similar to those used in the male rat experiment. Ghrelin resulted in a decrease of the BOLD response in all ROIs both in the OVX (n = 7) and OVX+E2 (n = 7) groups. The characteristics of the response (slope, duration, maximal effect) were almost identical in males and females as exemplified in the prefrontal cortex (Fig. 4A), nucleus accumbens (Fig. 4B), ventromedial nucleus (Fig. 4C) and the paraventricular nucleus (Fig. 4D). Seemingly, neither sex nor E2 replacement in OVX females had impact on the ghrelin-evoked BOLD responses in hedonic and homeostatic centers of energy balance.

### Effect of Rimonabant on the BOLD Response to Ghrelin

**Figure 5:**
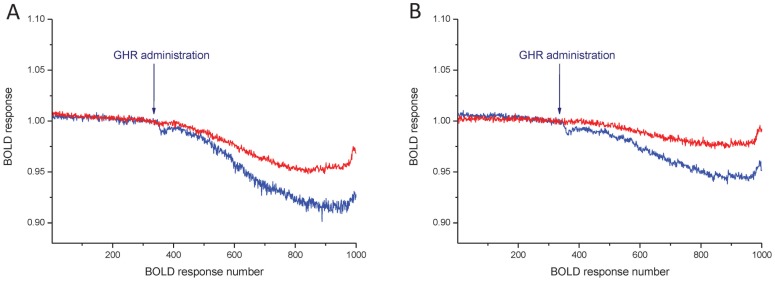
Mean of time response curves of ghrelin's effect on BOLD responses in the nucleus accumbens (A) and septum (B) after vehiculum (blue) or rimonabant pretreatment (red). Arrows mark ghrelin administration, which started at BOLD response 333 and lasted to 383 (999–1149 s). For quantifying drug effect, mean of the BOLD responses from 901 to 950 was calculated (2703–2850 s). N = 5–7.

**Figure 6:**
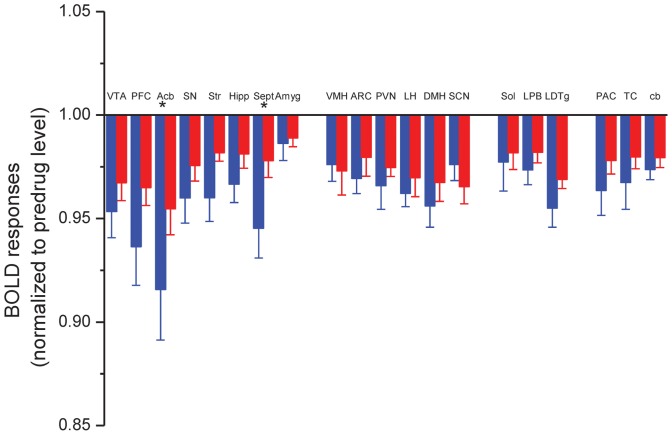
Effect of ghrelin on BOLD responses in reward processing centers, hypothalamic nuclei and brainstem areas. Effect of ghrelin on the BOLD responses was represented by the blue columns after vehicle pretreatment, and red columns after rimonabant pretreatment. Statistically significant differences were found in the nucleus accumbens and septum. ANOVA and Fisher *post hoc* test. *: p<0.05. N = 5–7.

The effect of CB1 receptor antagonist rimonabant (3 mg/kg, i.p.) was tested on the ghrelin response in the third experimental line of the study. The effect of rimonabant was evaluated in various areas by comparing BOLD responses to ghrelin in vehicle (n = 5–7) and rimonabant (n = 7) pretreated animals. Rimonabant highly and significantly attenuated the response to ghrelin in the nucleus accumbens (Fig. 5A) and septum (Fig. 5B). In other ROIs, rimonabant pretreatment did not interfere with the ghrelin-evoked BOLD response in a statistically significant manner (Fig. 6).

## Discussion

In this study, we explored the BOLD effects of peripherally administered ghrelin in hedonic and homeostatic centers regulating energy balance in the rat using fMRI. Ghrelin administered to the periphery can act on the brain directly [22], [47], or indirectly via vagal afferent [48]. We found a significant BOLD decrease in response to ghrelin in homeostatic feeding centers of the hypothalamus including the ventromedial nucleus, the paraventricular nucleus and the lateral hypothalamic area by comparing the saline and GHS-R1A antagonist pretreated animal groups. Similar significant responses were measured in reward signal processing structures of the telencephalon like the prefrontal cortex, the nucleus accumbens and the septum. These findings are in agreement with recent fMRI studies demonstrating ghrelin-evoked modulation of the brain reinforcement system [49] and key appetite regulatory regions of the hypothalamus [50] in rodents. In addition to the male, we also followed the BOLD response to ghrelin in females, using low (ovariectomized) and high (ovariectomized-E2 replaced) experimental E2 paradigms. The results do not support any sex difference in the ghrelin-evoked BOLD responses or the regulation of the response by the female gonadal hormone, E2. We note that in the male-female comparison we used OVX females with/without E2 replacement and compared them to intact males. For revealing the putative contribution of the endocannabinoid signaling mechanism to the ghrelin-evoked BOLD response, we also examined the effects of ghrelin administration in CB1 receptor blocked male rats and found interaction between the two signaling systems in telencephalic structures. From the results we conclude that i) ghrelin regulates components of the classic mesolimbic reward system, especially the prefrontal cortex, the nucleus accumbens and the septum; ii) ghrelin acts in key hypothalamic feeding centers, the ventromedial nucleus, the paraventricular nucleus and the lateral hypothalamic area; iii) the pattern of BOLD response to ghrelin resembles partly the published expression loci of GHS-R1A mRNA; iv) the effect of ghrelin on BOLD response depends on CB1 signaling in the nucleus accumbens and the septum; and v) ghrelin-evoked BOLD response is independent of sex and the actual E2 *milieu*.

### Ghrelin Modulates the BOLD Response of Reward Signal Processing Systems

The ventral tegmental area is a key center in reward processing [51]. It contains the A10 dopaminergic cell group [52] that gives rise to ascending mesocortical and mesolimbic projections that innervate the prefrontal cortex and the nucleus accumbens, respectively [53]. GHS-R1A mRNA has been detected within A10 dopaminergic neurons and cholecystokinin-immunoreactive neurons of the VTA [4]. Ghrelin regulates dopaminergic neurons in the VTA [54]. Via modulation of the mesolimbic dopaminergic system, ghrelin is capable of increasing food intake [14], [21], [23], modulating hedonic reward value of food, alcohol and addictive chemical substances [22], regulating hyperlocomotion caused by psychostimulant drugs [29], [55] and increasing effectiveness of learning and memory [56]–[58]. Our present findings are in concert with these physiological data by demonstrating that peripheral ghrelin administration causes marked alterations of the BOLD response in different structural constituents of the mesolimbic and mesocortical systems. Most notably, it modifies the BOLD response of the nucleus accumbens and prefrontal cortex in a significant manner. The effectiveness of JVM2959 treatment strengthens the view that the manifestation of the ghrelin-evoked BOLD response requires the contribution of GHS-R1A. The facilitative role of GHS-R1A in the regulation of the brain reinforcement system has recently been proposed in the rat based on pharmacological MRI analysis and intracranial self-stimulation [49]. Ghrelin increased the cerebral blood volume (rCBV) in medial and lateral hypothalamic structures and also in mesolimbic reward units, the ventral tegmental area, the nucleus accumbens and septum. The responsiveness of hypothalamic feeding nuclei to ghrelin has also been published in mice using manganase-enhanced MRI [50].

### Ghrelin Changes the BOLD Response of Homeostatic Feeding Centers in the Hypothalamus

GHS-R1A is extensively expressed in the rodent hypothalamus [3]–[5], [59], [60]. In concert with the distribution pattern of GHS-R1 mRNA expression, we report matching BOLD response from the ventromedial nucleus, the paraventricular nucleus and the lateral hypothalamus. The evoked BOLD effects were attenuated by JMV2959. The arcuate nucleus did also respond to ghrelin, but the effect did not reach statistical significance. The orexigenic effects of ghrelin involve and require AMPK signaling in both the VMH [61] and the PVN [39], [62]. In the lateral hypothalamic area, ghrelin evokes Fos immunoreactivity in orexin neurons [63] and also inhibits their electrophysiological activity [9]. Binding of iodinated ghrelin ligand has been reported [6] in all feeding centers of the hypothalamus that showed BOLD response to ghrelin challenge in our study.

### Pharmacological Blocking of CB1 Signaling Attenuates the Ghrelin-Evoked BOLD Response

Both ghrelin and endocannabinoids regulate energy balance [64], [65]. The ghrelin and endocannabinoid signaling mechanisms are interrelated in the hypothalamus. Blocking CB1 receptor by specific antagonists abolishes the orexigenic effect of ghrelin [66]. Ghrelin is also incapable of triggering orexigenic effects in CB1-knockout mice [39]. Activation of GHS-R has been shown to increase the endocannabinoid content of the hypothalamus [39]. Ghrelin inhibits the excitatory inputs to parvocellular paraventricular neurons [39] and gonadotropin-releasing hormone (GnRH) neurons [40] via the cooperation with the endocannabinoid-CB1 system. While the individual regulatory capacity of both the endocannabinoid and ghrelin systems has been confirmed in other parts of the brain as well [67]–[69], the coupled nature of these signaling mechanisms awaits further studies and clarification. In our fMRI study, the pharmacological inhibition of CB1 by rimonabant abolished the BOLD response to ghrelin in the nucleus accumbens and the septum. This result is in concert with the finding that 2-arachidonoylglycerol signaling through CB1 regulates the activity of forebrain neural circuits that control energy expenditure [70]. The BOLD response of the hippocampus to ghrelin was only partially attenuated by rimonabant. In individual hypothalamic nuclei, the blockade of CB1 receptor did not counteract the BOLD effects of ghrelin. Seemingly, this finding is in conflict with physiological data exploring the coupled nature of ghrelin and endocannabinoid signaling mechanisms. It needs further studies, although, it is also possible that the current resolution power of the used fMRI technology does not allow the imaging of this particular molecular interaction in small-sized hypothalamic nuclei.

### Correlating Human and Rodent fMRI Data about the Central Effects of Ghrelin

The current data and the previously reported fMRI results [49], [50] on actions of ghrelin in the CNS are in consensus and suggest that ghrelin exerts the most potent effects on the mesolimbic reward system (VTA, Acb, PFC, Sept) and hypothalamic centers engaged to regulation of feeding and energy expenditure (ARC, VMH, PVN, LH, SCN). Therefore, in rodents, fMRI offers a unique and powerful tool for the exploration of neuronal centers serving the hedonic and homeostatic components of feeding under the challenge of orexigenic and anorexigenic hormones. In humans, ghrelin was reported to modulate the activity of brain regions involved in the control of appetitive behavior [71]. BOLD changes characterized visual information processing centers (occipital gyrus, fusiform gyrus), the insula, as the main gustatory center and limbic structures (substantia nigra, striatum, orbitofrontal cortex, amygdala, caudate and hippocampus) involved in appetite behavior. Fasting levels of ghrelin also correlate with the brain response to pictures of palatable food, with activation of visual, gustatory, limbic reward and hypothalamic centers [72]. A bolus injection of ghrelin in the postprandial period decreases the BOLD response of several brain regions known to control appetite and feeding in humans and it inhibits the response of the CNS to ingested lipid [73]. In the latter study, the response remained essentially unchanged after entering the covariates of body mass index and sex. Our study, also favors the lack of sex specificity of ghrelin action in the rodent brain.

In summary, the present study revealed that ghrelin evokes a marked response in the rodent brain and modulates both the hedonic and homeostatic components of feeding regulating neuronal assemblies. The generated response is not sex specific and the manifestation of ghrelin's effect depends on endocannabinoid signaling in main reward signal processing centers. Furthermore, at the level of CNS, the responses to ghrelin are remarkably similar in humans and rats.
